# Can the Accuracy of Home Blood Glucose Monitors be affected by the Received Signal Strength of 900 MHz GSM Mobile Phones?

**Published:** 2015-12-01

**Authors:** J. Eslami, F. Ghafaripour, S.A.R. Mortazavi, S.M.J. Mortazavi, M.B. Shojaei-fard

**Affiliations:** 1 Lecturer of Anesthesiology, Anesthesiology Department, School of Nursing and Midwifery, Shiraz University of Medical Sciences, Shiraz, Iran; 2 Medical Physics & Medical Engineering Department, School of medicine, Shiraz University of Medical Sciences, Shiraz, Iran; 3 Medical Student, School of medicine, Shiraz University of Medical Sciences, Shiraz, Iran; 4 Department of Physiology, Fasa University of Medical Sciences, Fasa, Iran; 5 Ionizing and Non-ionizing Radiation Protection Research Center, Shiraz University of Medical Sciences, Shiraz, Iran

**Keywords:** Blood Glucose Self-Monitoring, Radio Waves, Cell Phones, Medical Device Safety

## Abstract

**Background:**

People who use home blood glucose monitors may use their mobile phones in the close vicinity of medical devices. This study is aimed at investigating the effect of the signal strength of 900 MHz GSM mobile phones on the accuracy of home blood glucose monitors.

**Methods:**

Sixty non-diabetic volunteer individuals aged 21 - 28 years participated in this study. Blood samples were analyzed for glucose level by using a common blood glucose monitoring system. Each blood sample was analyzed twice, within ten minutes in presence and absence of electromagnetic fields generated by a common GSM mobile phone during ringing. Blood samples were divided into 3 groups of 20 samples each. Group 1: exposure to mobile phone radiation with weak signal strength. Group2: exposure to mobile phone radiation with strong signal strength. Group3: exposure to a switched–on mobile phone with no signal strength.

**Results:**

The magnitude of the changes in the first, second and third group between glucose levels of two measurements (׀ΔC׀) were 7.4±3.9 mg/dl, 10.2±4.5 mg/dl, 8.7±8.4 mg/dl respectively. The difference in the magnitude of the changes between the 1st and the 3rd groups was not statistically significant. Furthermore, the difference in the magnitude of the changes between the 2nd and the 3rd groups was not statistically significant.

**Conclusion:**

Findings of this study showed that the signal strength of 900 MHz GSM mobile phones cannot play a significant role in changing the accuracy of home blood glucose monitors.

## Introduction

Mobile phones are modern communication devices which are being used for a variety of purposes, including contacting with family members, conducting business and  accessing  emergency services in a critical situation .They can also have advantages for patient care and can improve communication and data access [1]. Mobile phones use electromagnetic radiation in microwave range [2]. In Iran digital mobile phones currently operate at frequencies around 900MHz with single-band technology and 900MHz / 1800MHz with dual-band technology. Therefore in a specific time it cannot be determined which frequency is being used.

Although mobile phone use in hospitals is restricted due to the risk of interference with medical equipments, people who use home blood glucose monitors may use their mobile phones in the close vicinity of these devices. The immunity level for non-life supporting medical devices at present for radiated RF is 3V/m, although in some cases electromagnetic radiation (EMR) produced by mobile phones could reach  42 V/m at 0.1 m [3, 4].

Power of mobile phones is not fixed and it changes with distance and quality of signal received from the base stations. Therefore when the network coverage is weak mobile phones transmit power may reach the maximum levels that can increase the risk of Electromagnetic Interference (EMI) [5].

The response of a medical device with EMI caused by a mobile phone depends on many factor like characteristics of the waveform (frequency, modulation), other external signals, the power of the signal, the environment in which the medical device is operating and device immunity level [5].

The increased use of mobile phones throughout the world has raised concerns about their potential ability to cause EMI in medical devices. Over the past several years different researchers have reported EMI induced by mobile phones in medical devices [6-16]. EMFs produced by mobile phones are capable of altering analog signal or digital data in susceptible medical devices which can lead to inaccurate results or even degradation in device normal function [17].

Controlling blood sugar is an important part of managing diabetes. Blood glucose monitoring devices are test systems which can be used at home to measure the amount of glucose in human blood. This quantitative test can help diabetic patients and physicians to determine diabetic patient daily adjustments in treatment, assessment of glucose level to determine high risk patients, understand the effect of diet and exercise on glucose levels and finally good glucose control using home monitors can lead to fewer disease complication [18].

Over the past several years, our laboratories have expanded their focus on studying the health effects of exposure to some common and/or occupational sources of EMFs such as cellular phones [19-26], mobile base stations [27], mobile phone jammers [28], laptop computers [29], radars [20], dentistry cavitrons [30] and Magnetic Resonance Imaging (MRI) [25, 31]. We have previously shown that EMI from mobile phones can adversely affect the accuracy of home blood glucose monitors. It was suggested by our team that mobile phones should be used at least 50 cm away from home blood glucose monitors [32]. The aim of this study was to investigate whether the signal strength of 900 MHz GSM mobile phone can affect the accuracy of home blood glucose monitors.

## Materials and Methods

This study was conducted over a period of 3 month from October to December 2014 in Namazi Hospital after obtaining ethical clearance from INIRPRC (Ionizing and Non-ionizing Radiation Protection Research Center).  The selection of study participants was based on random sampling method. Sixty non-diabetic volunteer individuals aged 21 - 28 years participated in this study and in terms of gender they were equally distributed in each group. Inclusion criteria was apparent health and exclusion criteria was any self-reported disease. Eligible individuals were randomized into 3 groups of 20 each. The protocols and informed consent were reviewed and approved by INIRPRC. The study and its objectives were explained to all study participants and verbal informed consent was obtained before enrolment. Blood samples were analyzed for glucose level by using a new ACCU CHEK-Active (DBMnul002, Germany) blood glucose monitoring system. Blood samples were divided into 3 groups of 20 samples each. Samples in the 1^st^ group were exposed to the radiation emitted by the mobile phone with weak signal strength, while the signal strength in the 2^nd ^group was strong. The mobile phone in the 3^rd^ group was in standby mode but it had no signal strength. Each blood sample was analyzed twice, within ten minutes after blood sampling with lancet, in the presence or absence of electromagnetic fields caused by a common GSM mobile phone (Nokia, N73) during ringing.

**Figure 1:**
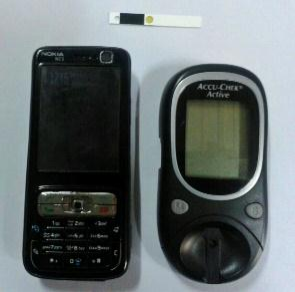
Position of the mobile phone and the ACCU CHECK home blood glucose monitor during measurement.

The position of cell phone and home blood glucose monitor during measurement is shown in Figure 1. At the end of measurement in designated groups, the magnitude of changes in between glucose levels (׀ΔC׀) in presence and absence of EMF was calculated.

ΔC= Glucose level in the 1^st^ measurement - Glucose level in the 2^nd^ measurement

In this equation, glucose level in the 2^nd^ measurement was the measurement in the presence of EMF with different signal strength.

### Statistical analysis

Data were analyzed using SPSS (IBM SPSS Statistic 19). Basic data were described by mean, min, max, SD and delta for numeric data. Deltas of test groups were assessed with a One-Way ANOVA test. P values less than 0.05 considered as significant. 

## Results

Blood glucose levels (mg/dl) in 20 participants of the 1^st^ group whose glucose tests were performed in the presence of the electromagnetic radiation emitted by the mobile phone with weak signal strength are indicated in Table 1. Blood glucose levels in the 1^st^ group before and after exposure were 93.9 ± 8.5 mg/dl and 101.3 ± 11.3 mg/dl, respectively. The magnitude of the changes in this group between glucose levels of two repeated measurements (׀ΔC׀) was 7.4±3.9 mg/dl.

**Table 1:** Blood glucose levels (mg/dl) of the 1st group

	**age**	**Control** **1st measurement** **Glucose Level (mg/dl)**	**EMF** **2nd measurement** **Glucose Level (mg/dl)**	**ΔC** **(mg/dl)**	**|Delta|**
	22	94	104	10	10
	21	95	104	9	9
	27	85	89	4	4
	23	103	119	16	16
	22	95	108	13	13
	22	125	138	13	13
	23	96	101	5	5
	24	87	90	3	3
	24	91	96	5	5
	25	94	106	12	12
	24	96	101	5	5
	25	87	90	3	3
	24	92	98	6	6
	25	90	93	3	3
	25	88	94	6	6
	24	96	100	4	4
	24	92	103	11	11
	24	87	92	5	5
	25	95	103	8	8
	24	89	96	7	7
**Mean**	**23.85**	**93.85**	**101.25**	**7.4**	**7.4**
**SD**	**1.39**	**8.52**	**11.31**	**3.89**	**3.89**
**Min**	**21**	**85**	**89**	**3**	**3**
**Max**	**27**	**125**	**138**	**16**	**16**

Table 2 shows the blood glucose levels (mg/dl) in 20 participants of 2^nd^  group whose glucose tests were performed in the presence of the electromagnetic radiation emitted by the mobile phone with strong signal strength. Blood glucose levels in the 2^nd^ group before and after exposure were 93.5 ± 8.2 mg/dl and103.7 ± 6.2 mg/dl, respectively. The magnitude of the changes in this group between glucose levels of two repeated measurements (׀ΔC׀) was 10.2±4.5 mg/dl.

**Table 2:** The blood glucose levels (mg/dl) of 2nd  group

	**age**	**Control** **1st measurement** **Glucose Level (mg/dl)**	**EMF** **2nd measurement** **Glucose Level (mg/dl)**	**ΔC** **(mg/dl)**	**|Delta|**
	22	87	104	18	18
	23	92	104	12	12
	22	89	106	17	17
	21	89	103	14	14
	23	98	101	3	3
	22	105	109	4	4
	23	101	110	9	9
	21	119	123	4	4
	27	89	100	11	11
	28	92	100	8	8
	26	87	96	9	9
	27	101	108	7	7
	26	83	99	16	16
	27	88	105	17	17
	26	91	96	5	5
	26	89	100	11	11
	26	93	102	9	9
	25	87	96	9	9
	24	92	104	12	12
	22	97	107	10	10
**Mean**	**24.35**	**93.45**	**103.7**	**10.25**	**10.25**
**SD**	**2.28**	**8.24**	**6.20**	**4.50**	**4.50**
**Min**	**21**	**83**	**96**	**3**	**3**
**Max**	**28**	**119**	**123**	**18**	**18**

Finally blood glucose levels (mg/dl) in 20 participants of the 3^rd^  group whose glucose tests were performed in the close vicinity of mobile phone with no signal are indicated in Table 3. Blood glucose levels in the 3^rd^ group before and after exposure were 95.1 ± 8.3 mg/dl and 102.6 ± 12.0 mg/dl, respectively. The magnitude of the changes in this group between glucose levels of two repeated measurements (׀ΔC׀) was 8.7±8.4 mg/dl.

**Table 3:** Blood glucose levels (mg/dl) of the 3rd  group

	**age**	**Control** **1st measurement** **Glucose Level (mg/dl)**	**EMF** **2nd measurement** **Glucose Level (mg/dl)**	**ΔC** **(mg/dl)**	**|Delta|**
	20	90	92	2	2
	20	90	94	4	4
	22	97	109	12	12
	21	87	92	5	5
	21	98	101	3	3
	19	102	105	3	3
	21	97	109	12	12
	21	105	99	6	6
	23	83	84	1	1
	22	92	94	2	2
	22	85	102	17	17
	20	93	107	14	14
	20	99	103	4	4
	27	111	142	31	31
	21	88	91	3	3
	20	105	99	-6	6
	21	107	110	3	3
	20	101	110	9	9
	20	89	98	9	9
	21	83	111	28	28
**Mean**	**21.1**	**95.1**	**102.6**	**8.7**	**8.7**
**SD**	**1.68**	**8.33**	**12.01**	**8.40**	**8.40**
**Min**	**19**	**83**	**84**	**1**	**1**
**Max**	**27**	**111**	**142**	**31**	**31**

The difference in the magnitude of the changes between the 1^st^ and the 3^rd^ groups and also 2^nd^ and the 3^rd^ groups were not significant (p =0.32).Therefore Post-Hoc multiple comparisons were not used.

## Discussion

Findings of this study showed that the signal strength of 900 MHz GSM mobile phones cannot play a significant role in changing the accuracy of home blood glucose monitors. It is worth mentioning that we have previously reported that mobile phones as the most widely used communication devices may cause EMI in home blood glucose monitors. This kind of interference was observable when the mobile phone was in the talk mode. Our current study showed that the signal strength is not an important factor in inducing EMI in home blood glucose monitors. As the home blood glucose monitors used in these two studies are different (BIONIME ,GM110, Taiwan in our previous study vs. ACCU CHEK-Active, DBMnul002, Germany in our present study), the observed difference in the susceptibility of these devices may be due to their different manufacturing characteristics. 

With regard to rapidly increasing number of individuals who use home blood glucose monitors, identification of different possible sources of EMI is a growing need. As numerous factors are involved in the potential interferences (factors such as geometry of the exposure, output power of cell phone, antenna characteristics and the distance between the cell phone and the nearest base station) users should be encouraged to keep a safety distance between their cell phones and home blood glucose monitors to prevent any possible interference. 

To the best of our knowledge, this is the first study to assess the role of signal strength received by mobile phones in potential EMI of home blood glucose monitors. As mobile phone communication can play an increasingly important role in efficient patient healthcare, the use of this communication device cannot be totally prohibited in the vicinity of medical devices. However, setting effective guidelines about their usage and enhancement of the immunity of medical devices can decrease the incidence of EMI in medical devices. It can be concluded that during blood glucose measurement it is advisable to place mobile phones away from any susceptible measurement device to prevent inaccuracy in device readout. Using only one brand/model of mobile phone and home blood glucose monitor was the main limitation of our study. Further experiments are needed to clarify different aspects of the susceptibility of home blood glucose monitors to electromagnetic interference. 

## Conclusion

Based on the results obtained in this study it can be concluded that mobile phones signal strength cannot significantly alter the accuracy of home blood glucose monitors. More studies using blood glucose monitors and mobile phones made by different manufactures are needed to better understand the role of mobile phone signal strength on the occurrence of EMI. 
